# Circulating miR-26b-5p and miR-451a as diagnostic biomarkers in medullary thyroid carcinoma patients

**DOI:** 10.1007/s40618-023-02115-2

**Published:** 2023-06-07

**Authors:** Z. M. Besharat, S. Trocchianesi, A. Verrienti, R. Ciampi, S. Cantara, C. Romei, C. Sabato, T. M. R. Noviello, A. Po, A. Citarella, F. P. Caruso, I. Panariello, F. Gianno, G. Carpino, E. Gaudio, M. Chiacchiarini, L. Masuelli, M. Sponziello, V. Pecce, T. Ramone, F. Maino, F. Dotta, M. Ceccarelli, L. Pezzullo, C. Durante, M. G. Castagna, R. Elisei, E. Ferretti

**Affiliations:** 1 Department of Experimental Medicine, Sapienza University of Rome, Viale Regina Elena 324, 00161 Rome, Italy; 2 Department of Molecular Medicine, Sapienza University of Rome, 00161 Rome, Italy; 3 Department of Translational and Precision Medicine, Sapienza University of Rome, 00161 Rome, Italy; 4 Endocrine Unit, Department of Clinical and Experimental Medicine, University of Pisa, 56126 Pisa, Italy; 5 Department of Medical, Surgical and Neurological Sciences, University of Siena, 53100 Siena, Italy; 6 Biogem Scarl, Istituto di Ricerche Genetiche “Gaetano Salvatore”, 83031 Ariano Irpino, Italy; 7 Department of Electrical Engineering and Information Technology, University of Naples Federico II, 80138 Naples, Italy; 8 Thyroid Surgical Unit, IRCCS Fondazione G.Pascale, 80131 Naples, Italy; 9 Department of Radiological, Oncological and Anatomo-Pathological Sciences, Sapienza University of Rome, 00161 Rome, Italy; 10 Department of Anatomical, Histological, Forensic Medicine and Orthopedics Sciences, Sapienza University of Rome, Rome, Italy; 11 Tuscany Centre for Precision Medicine (CReMeP), 53100 Siena, Italy

**Keywords:** Medullary thyroid carcinoma, Diagnostic biomarkers, miR-26b-5p, miR-451a, Liquid biopsy, Plasma extracellular miRNAs/circulating miRNAs

## Abstract

**Purpose/methods:**

The determination of tumour biomarkers is paramount to advancing personalized medicine, more so in rare tumours like medullary thyroid carcinoma (MTC), whose diagnosis is still challenging. The aim of this study was to identify non-invasive circulating biomarkers in MTC. To achieve this goal, paired MTC tissue and plasma extracellular vesicle samples were collected from multiple centres and microRNA (miRNA) expression levels were evaluated.

**Results:**

The samples from a discovery cohort of 23 MTC patients were analysed using miRNA arrays. Lasso logistic regression analysis resulted in the identification of a set of circulating miRNAs as diagnostic biomarkers. Among them, miR-26b-5p and miR-451a, were highly expressed and their expression decreased during follow-up in disease-free patients in the discovery cohort. Circulating miR-26b-5p and miR-451a were validated using droplet digital PCR in a second independent cohort of 12 MTC patients.

**Conclusion:**

This study allowed the identification and validation of a signature of two circulating miRNAs, miR-26b-5p and miR-451a, in two independent cohorts reporting a significant diagnostic performance for MTC. The results of this study offer advancements in molecular diagnosis of MTC proposing a novel non-invasive tool to use in precision medicine.

**Supplementary Information:**

The online version contains supplementary material available at 10.1007/s40618-023-02115-2.

## Introduction

Medullary thyroid carcinoma (MTC) is a rare tumour originating from thyroid parafollicular C cells and can be hereditary in 20–25% or sporadic in 75–80% of cases [1]. Mutations of REarranged during Transfection (RET) proto-oncogene are common in hereditary MTC and can also characterize sporadic MTC along with RAS mutations [2, 3]. MTC patients’ outcome depends on the extent of the disease. In detail, 75% will present nodal metastasis while less than 10% present metastatic spread at diagnosis [4]. Approximately 30% of patients without detectable metastases at diagnosis will not be cured by surgery and will present progressive disease with the appearance of metastases during the follow-up. Overall, considering all MTC patients, about 50% will recur with poor outcome [5].

MTC diagnosis is performed by thyroid nodule biopsy and assessment of calcitonin (Ctn) is mandatory either through immunohistochemical positivity for Ctn or measurement of Ctn from fine needle aspiration biopsies and deregulated Ctn serum levels. Of note, elevated Ctn serum levels are not only associated to MTC but also to non-tumoral conditions such as C cell hyperplasia, goiter and renal insufficiency as well as in a few non-thyroid related tumours (neuroendocrine tumours of the lung or gastro­intestinal tract) [6–8]. MTC patients follow-up is performed by assessment of serum Ctn and carcinoembryonic antigen (CEA) along with imaging evaluations [9], however MTC progression is not always accompanied by the increase of these serum biomarkers leading to MTC patients’ progression remaining undetected until the presence of a new lesion.

In this context, to identify new molecular biomarkers of MTC we focused our attention to plasma extracellular vesicles (pEVs) as a new source of circulating molecules [10–12]. PEVs are secreted into the blood stream by cells and transport microRNAs (miRNAs) along with other cellular factors [13].

MiRNAs are a class of small non-coding RNAs able to regulate gene expression at a post-transcriptional level [14, 15]. MiRNAs have been associated with different clinical conditions and their expression patterns in MTC tumour samples have been the object of investigation [16, 17]. Circulating miRNAs satisfy several criteria for being considered as good biomarkers, such as a non-invasive procedure for their detection, being robust, easy to detect with different technologies, affordable and whose detection can be performed in a short time while providing a snapshot of what is happening in the organism since different types of cells like tumour cells, cells of the immune system and cells of the microenvironment secrete miRNAs. On this basis, the deregulation of circulating miRNAs in cancer has prompted their use as diagnostic, prognostic and response to treatment biomarkers [16, 18].

To date, three studies have described circulating miRNAs in MTC, specifically miRNAs from plasma or serum samples of MTC patients. In detail, authors investigated the expression of selected miRNAs and reported their high expression compared to healthy controls, namely miR-375 [19], miR-144 and miR-34a [20] and miR-222-3p and miR-17-5p [21].

Interestingly, there is no report describing circulating miRNA profiles from MTC patients and more importantly no report presenting plasma-EV (pEV) MTC miRNA profiles.

The aim of this study was to analyse tumour miRNA and circulating pEV miRNA profiles of MTC patients to identify new biomarkers. The results of this study allowed the identification of pEV miR-26b-5p and miR-451a as diagnostic and monitoring biomarkers.

## Materials and methods

**Fig. 1:**
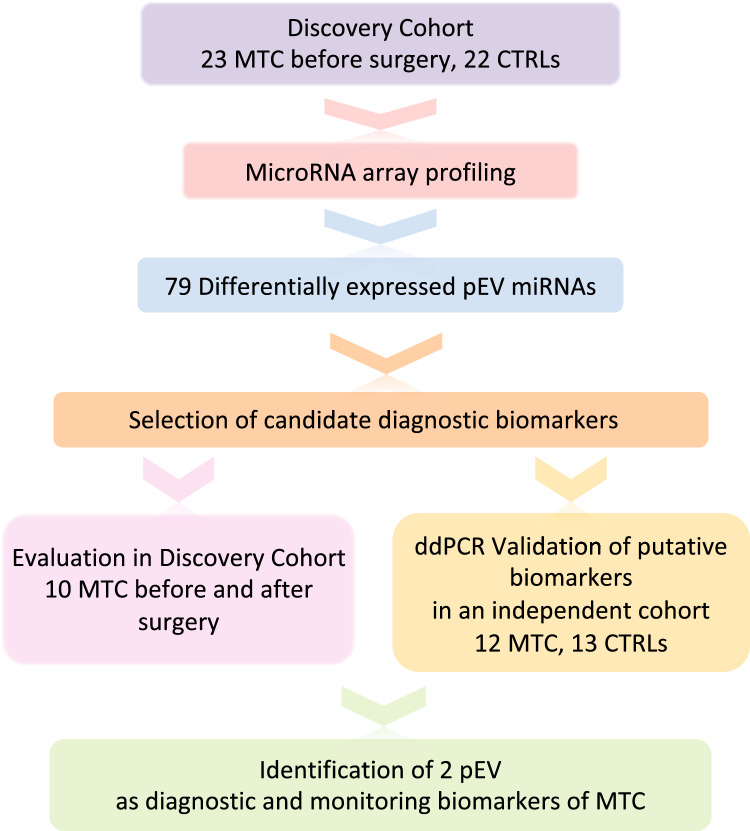
Flow diagram of the study describing the steps that led to the identification of circulating extracellular vesicle miRNAs as diagnostic and monitoring biomarkers of medullary thyroid carcinoma

A workflow of the study describing the steps that led to the identification of the circulating pEV miRNAs as diagnostic biomarkers of MTC is shown in Fig. 1.

### Discovery and validation cohorts

Medullary thyroid carcinoma (MTC) patients were enrolled in three different Italian centres: Department of Medicine, Surgery and Neuroscience at the University of Siena; Department of Clinical and Experimental Medicine at the University of Pisa; Thyroid Surgical Unit of the National Cancer Institute “Fondazione G. Pascale” in Naples. Exclusion criteria are listed below: patients with an undefined thyroid cancer diagnosis; patients with concomitant second malignant tumours in the last 5 years; patients treated with systemic therapies in the 6 months prior to enrolment; patients with hereditary MTC and patients aged < 18 years at diagnosis. Clinical data of MTC patients were included in a computerized database collecting epidemiological, medical, and pathological features. The discovery cohort consisted of 23 MTC patients and 22 control subjects.

Patients underwent surgery and 23 primary tumour tissues were collected along with their respective pre-operative whole blood samples. Enrolled MTC patients included 11 male and 12 female patients with a mean age of 54 years at diagnosis.

In addition, as control samples (CTRL), whole blood samples were collected from 22 healthy donors, that included 10 male and 12 female donors with a mean age of 46 years. Characteristics and clinical data for MTC and CTRL discovery cohorts are reported in Table 1 and Supplementary Table 1.

**Table 1:** Clinical characteristics of medullary thyroid carcinoma (MTC) patients of the discovery cohort

ID MTC patient	Sex	Age	Mutation status for RET/HRAS	Type of RET mutation	TNM classification	AJCC staging	Tumour size (mm)	Calcitonin pre-surgery (ng/L)	CEA pre-surgery (ng/L)	Calcitonin post-surgery (ng/L)	CEA post-surgery (ng/L)	Follow up status	Monitoring cohort
2	Female	51	RET	630	T1bN0M0	I	13	0.404	Not available	0.0009	1.9	Cured	X
4	Female	66	RET	634	T2N0M0	II	21	357	0.3073	< 2	0.0011	Cured	
5	Female	62	OTHER (HRAS Q61K)		T1bN0M0	I	11	331	0.0223	< 2	0.0021	Cured	
6	Male	62	RET	634	T1bN1aM0	III	19	126	0.0391	< 2	0.0047	Cured	
7	Female	50	RET	918	T2N1aM0	III	35	443	0.0111	37.1	0.0025	Structural disease under treatment	
8	Male	27	WT		T3N0M0	II	50	5843	0.2786	< 2	0.004	Cured	X
9	Female	38	RET	634	T1N1aM0	III	10	42.4	Not available	10.8	0.0021	Cured	X
12	Male	78	RET	620	T1bcN0cM0	I	15	84.7	Not available	9.3	Not available	Cured	
13	Female	59	RET	634	pT2cN0M0	I	21	683	Not available	0.9	Not available	Cured	
14	Female	43	RET	634	T2mN1BM1	II	22	171.5	204.5	172	Not available	Deceased	
15	Female	62	RET	918	T3bN1bM1	IVb	45	118	2.48	197	3.074	Deceased	
16	Male	49	WT		T2bN1bM1	II	30	512	Not available	3.26	0.0004	Structural disease	
17	Female	29	RET	918	T2N0M0	II	23	Not available	Not available	Not available	Not available	Not available	
18	Male	77	WT		T2N1bM1	IVc	37	1.992	0.15	1.205	184.9	Structural disease	X
19	Male	74	OTHER (HRAS)		T1bN0M0	I	17	1.322	38.4	0.0018	3.2	Cured	X
21	Male	72	OTHER (HRAS)		T1aN0M0	I	8	0.0907	Not available	< 1.0	0.0019	Cured	X
22	Female	63	OTHER (KRASG12R)		T1bN0M0	I	20	0.525	Not available	< 1.0	0.0011	Cured	X
23	Female	28	RET	634	T1bN0M0	I	11	0.0682	0.0088	3.3	0.0015	Cured	X
26	Male	41	RET	634	T1bN0M0	I	12	66.7	0.0074	< 2	0.0005	Cured	
29	Male	76	OTHER (HRAS G13R)		T3aN1aM0	III	47	5254	0.182	7.8	0.0048	Biochemical disease	X
30	Male	68	RET	630	T3aN1aM0	III	49	7838	0.029	437	0.0027	Biochemical disease	
31	Female	32	RET	902	T1bN0M0	I	Not available	311	0.0344	< 2	0.0015	Cured	
32	Male	49	RET	634	T2N0M0	II	23	251	0.665	< 2	0.0015	Cured	X

MTC patients (*n* = 23)	
Sex	
Male	*n* = 11
Female	*n* = 12
Age	
Mean	54.61 years
Median	59 years
Range	27–78 years
Mutation status for RET/HRAS	
RET positive	*n* = 15
RAS positive	*n* = 5
RET/RAS negative	*n* = 3
Follow up category	
Cured	*n* = 15
Structural disease under treatment	*n* = 1
Structural disease	*n* = 2
Biochemical disease	*n* = 2
Deceased	*n* = 2
Not available	*n* = 1

A second independent cohort was enrolled as a validation cohort comprising of 12 MTC subjects and 13 CTRL subjects. The validation cohort included 4 male and 8 female with a mean age of 50 years for MTC patients and 8 male and 5 female with a mean age of 47 years for CTRL subjects. Characteristics and clinical data for the validation cohort are reported in Table 2 and Supplementary Table 1.

**Table 2:** Clinical characteristics of medullary thyroid carcinoma (MTC) patients of the validation cohort

ID MTC patient	Sex	Age	Mutation status for RET/HRAS	Type of RET mutation	TNM classification	AJCC staging	Tumour size (mm)	Calcitonin pre-surgery (ng/L)	CEA pre-surgery (ng/L)	Calcitonin post-surgery (ng/L)	CEA post-surgery (ng/L)	Follow up status
50	Male	70	Not available		Not available	Not available	Not available	242	45.13	Not available	Not available	Not available
49	Female	45	OTHER (HRAS)		T1aN0M0	I	7	251	66.5	< 2	1.5	Biochemical disease
48	Female	44	RET		pT1bN0M0	I	20	523	0.0259	< 1.0	0.0021	Cured
47	Female	54	RET		pT3N1bM0	I	10	3230	2.53	Deceased	Deceased	Deceased
46	Male	40	RET		pT1aN0M0	I	10	70.1	0.0048	13.4	0.0016	Biochemical disease
45	Female	73	WT		pT1b(m)N0M0	I	10	346	0.0577	< 1.0	0.0044	Cured
44	Male	36	RET		pT1bN1bM0	I	10	15,600	0.0515	244	0.0073	Biochemical disease
43	Female	49	OTHER (HRAS Q61R)		T1aN1aM0	III	7	17.2	< 0.002	1.4	0.001	Cured
42	Female	56	OTHER (KRAS G12R)		T2N1aM0	III	28	1750	0.0747	11.2	0.0031	Biochemical disease
37	Female	59	OTHER (HRAS Q61R)		T1bN1aM0	III	12	69.1	0.0017	< 2	0.0016	Cured
38	Male	43	RET	634	T1bN0M0	I	15	99.9	0.0218	< 2	0.0013	Cured
40	Female	32	RET	918	T1bN0M0	I	15	127	0.0112	< 2	0.0014	Cured

MTC patients (*n*=12)	
Sex	
Male	*n*=4
Female	*n*=8
Age	
Mean	50.08 years
Median	47 years
Range	32–73 years
Mutation status for RET/HRAS	
RET positive	*n*=6
RAS positive	*n*=4
RET/RAS negative	*n*=1
Not available	*n*=1
Follow up category	
Cured	*n*=6
Structural disease under treatment	*n*=0
Structural disease	*n*=0
Biochemical disease	*n*=4
Deceased	*n*=1
Not available	*n*=1

Informed written consent was obtained before enrolment, according to the ethical committee guidelines (Protocol: OTC-CBSS-1114, Ethical committee reference: 4940).

### Discovery cohort MTC patients’ follow-up

MTC patients from the discovery cohort were monitored after surgery and plasma samples from 10 MTC patients were collected 3 months after surgery during follow-up to evaluate pEV miRNA expression levels as described above. MTC patients were classified 3 months after surgery based on whether they presented evidence of disease. In detail, 3 months after surgery 2 patients presented evidence of disease, while 8 were classified as disease free (as reported in Table 1).

### Tumour samples

All MTC tumour samples, of which 7 fresh-frozen (FF) and 16 formalin-fixed paraffin-embedded (FFPE), along with the MTC blood samples from the three Italian centres, were sent to the Department of Experimental Medicine, Sapienza University of Rome for the study.

### RNA extraction from MTC tumour samples

Two slides were cut by microtome from each FFPE block; one hematoxylin–eosin (H&E) slide was reviewed by a specialist pathologist to identify the tumour and comment on cellularity and tumour content, and one 10 µm non-stained slide was used for RNA extraction. The marked H&E slide was used as a guide for manual microdissection of tissue from the non-stained slides.

RNA was extracted from both FFPE and FF samples using Norgen Tissue Samples FFPE RNA Extraction Kit (#25300) and Trizol (ThermoScientific) respectively, according to manufacturer’s instruction.

Spike-in (Ath-miR159a), 5 μl of 400 pM solution, was added to each sample and expression of spike-in was evaluated before miRNA profiling as technical quality check. The extracted RNA was eluted in 30 μl of H_2_O RNase-free and quantity and quality were evaluated with a Nanodrop ND-100 spectrophotometer (ThermoScientific).

For each sample, retrotranscription was performed using as input 10 ng of total RNA for TaqMan^®^ Advanced miRNA cDNA Synthesis Kit (Applied Biosystems, ThermoScientific).

### Plasma collection

Whole blood samples from MTC patients and control subjects (CTRL) were collected in EDTA tubes. From each subject, blood samples were processed by serial centrifugations: at 1300×*g* for 10 min at room temperature (RT), at 1200 g for 20 min at RT and finally at 10,000 g for 30 min at RT. Plasma fraction was transferred to RNase-free tubes and stored at − 80 °C until RNA extraction.

### Plasma extracellular vesicle (pEV) isolation

For defibrination of plasma samples 8ul of Thrombin (Ci = 611 U/ml) (System Biosciences) were added to each mL of plasma. After centrifugation at 10,000 RPM for 10 min at 4 °C the supernatant was transferred in new Eppendorf tube without disturbing the pellet to proceed with extracellular vesicles (EV) isolation. EVs were isolated using Exoquick (System Bioscience #EXOQ5A-1) following manufacturer's instructions. The EV pellet was resuspended in 200 µl of H_2_O RNase-free.

### pEV western blot analysis

Resuspended pEVs were lysed in RIPA buffer and processed as previously described [22]. Western blot analysis was performed using the following antibodies: CD63 (VPA00798; BioRad), Calnexin (sc-46669; Santa Cruz Biotechnology), HSP70 (sc-33575; Santa Cruz Biotechnology), TSG101 (HPA-006161; Atlas Antibodies), CD81 (sc-166029; Santa Cruz Biotechnology) as previously described [23]. The proteins were visualized on the BioRad ChemiDoc MP Imaging System (BioRad, Hercules, CA). The different molecular weights observed for TSG101 could be ascribed to post-translational modifications [24] and the different nature of the samples (whole cell lysate, pEVs) that were used.

### RNA extraction from pEV samples

RNA was extracted using PROMEGA Maxwell RSC miRNA Plasma and Serum kit (#AS1680) by the Maxwell^®^ RSC Instrument according to manufacturer’s instruction.

Extracted RNA was eluted in 30 μl of H_2_O RNase-free. Prior to RNA extraction, 5 μl of 400 pM solution of spike-in (Ath-miR159a) were added to each sample.

For each sample, 2 μl of RNA were used as input for TaqMan^®^ Advanced miRNA cDNA Synthesis Kit (Applied Biosystems, ThermoScientific).

Haemolysis evaluation was performed in plasma samples using the ratio of miR-451a to miR-23a-3p [delta Ct (miR-23a-3p—miR-451a)], by real-time reverse transcription (RT)-PCR using ViiA 7 Real-Time PCR System (ThermoScientific). Haemolysis was absent from all samples that were included in the study.

### MiRNA profiling in MTC tumour tissues and pEVs

MiRNA expression profiling was performed on tissue MTC and plasma samples using RT-qPCR with TaqMan Advanced miRNA Human A and B cards (Applied Biosystems, ThermoScientific), which detect 754 members of the human microRNA genome. Each reaction was performed according to Applied Biosystems protocols.

### MiRNA expression analysis

Analysis of tumour tissue and pEV miRNA expression levels was performed using R environment (http://www.r-project.org/). Data were cleaned, filtered, normalized and expression analysis was performed using the Bioconductor package HTqPCR [25]. Specifically, the RT-PCR cycle threshold, Ct, that were defined from the run as “Undetermined” were assigned a value of “Ct = 40”. Tumour tissue and pEV miRNA expression levels were obtained for each MTC sample using Ct values. Tumour tissue and pEV miRNA with Ct values > 33 were considered as not expressed. Tumour tissue and pEV miRNA with Ct values < 33 were considered informative and included in subsequent data expression analysis. Data were normalized using the quantile method for all tumour tissue and pEV samples.

Differential expression analysis was performed between MTC and CTRLs of the discovery cohort. pEV miRNAs with fold change >|1| and *p* < 0.05 were considered as differentially expressed (DE). DE pEV miRNAs were used as input data for hierarchical clustering. Clustering and heatmaps were also generated in R using the heatmap3 function.

### Identification of candidate pEV miRNA biomarkers in MTC patients

Penalized logistic regression was performed on pEV miRNA profiles of discovery cohort to determine the best pEV miRNA predictors of MTC status using the gmlnet R package [26]. The model was built on Ct values using DE pEV miRNAs. The least absolute shrinkage and selection operator (LASSO) regularization was applied to find a pEV miRNA signature minimizing the number of features. Logistic regression with the resulting signature was used to classify subjects with MTC.

Sensitivity, specificity, overall classification accuracy and area under the Receiver operating characteristics (ROC) curve were computed to assess classification performances using GraphPad Prism software Version 8 (La Jolla, California, USA).

Further expression and statistical significance criteria were applied on the four up-regulated miRNAs of the 13 miRNA signature obtained by the LASSO analysis. The Ct thresholds were defined based on the 13 pEV and tissue microRNA expression levels in all MTC samples. In detail, the high expression threshold was defined based on the 25% percentile of the microRNA values (Ct < 24), while the low expression level on the mean value minus the standard error of mean (Ct > 27). The criteria consisted of pEV miRNAs with high expression in MTC patients (low Ct values; Ct < 24), miRNA detection in at least 70% of the samples in each subject group and exclusion of those miRNAs expressed at low levels in MTC tumour samples (high Ct values; Ct > 27).

### In situ hybridization

In situ hybridization (ISH) was performed on sections (5 μm thick) cut from paraffin-embedded tissue blocks using ACD’s RNAscope ISH Technology—Red (ref. 3245), according to the manufacturer’s instruction. Hybridization was performed using U6 (727871-S1), Scramble (727881-S1), miRNAscope™ Probe SR-hsa-miR-451a-S1 (1125561-s1) and miRNAscope™ Probe SR-hsa-miR-26b-5p (1006321-S1). Slides were counterstained with Gill I Hematoxylin Sigma-Aldrich^®^ (GHS132-1L). Slides were scanned by a digital scanner (Aperio Scanscope CS System, Aperio Digital Pathology, Leica Biosystems, Milan, Italy) and processed by ImageScope.

### MiRNA enrichment analyses

MiRNA target determination was performed for miR-26b-5p and miR-451a using DIANA Tools mirPath v.3 [27]. Target genes were used as input for enrichment disease analysis and the top categories, containing the highest number of genes involved, are reported.

### Droplet digital PCR (ddPCR)

RNA was retrotranscribed using TaqMan^®^ Advanced miRNA cDNA Synthesis Kit omitting the pre-amplification step. The resulting cDNA was diluted 1:5 for miR-26b-5p, and a serial dilution of 1:5 and 1:100 for miR-451a, and 5 μl were used to prepare a 22 μl reaction mix containing 11 μl of 2X ddPCR Supermix for Probes (Bio-Rad) and 1,1 μl 20X TaqMan miRNA PCR primer probe set (#A25576 assay ID: 478418_mir for hsa-miR-26b-5p, assay ID: 478107_mir for hsa-miR-451a) (Life Technologies). The PCR mixes for each sample were loaded in a disposable cartridge (Bio-Rad) together with 70 μl of droplet generation Oil (Bio-Rad) and loaded in the QX200 droplet generator (Bio-Rad).

40 μl of droplets were then transferred into a 96 well plate and an endpoint PCR was performed using the following conditions: 95 °C for 10 min, then 45 cycles of 95 °C for 15 s and 58 °C for 1 min, and a final step at 98 °C for 10 min. Then, the 96 well plate was placed in the QX200 Droplet Reader for detection of positive droplets. The quantification of positive droplets was performed using the QuantaSoft software (Bio-Rad).

### Statistical analysis

Statistical analyses for ddPCR were performed using t-test for unpaired data (GraphPad Prism software Version 8, San Diego, California, USA).

ROC curves for the miRNAs of interest were performed using GraphPad Prism version 8 (San Diego, California, USA) and the Area under the ROC curve (AUC) was calculated. Correlation analyses for each pEV miRNA, miR-26b-5p and miR-451a, and Ctn levels after surgery and CEA levels after surgery collected during follow-up of the discovery cohort were performed using GraphPad Prism version 8 (San Diego, California, USA).

Multivariate analysis was performed for pEV miR-26b-5p and miR-451a using IBM SPSS Statistics v.27 (Armonk, New York, USA). Expression levels obtained from the validation cohort for each pEV miRNA were used as a dependent variable, Group (MTC or CTRL) was used as a fixed factor and sex, age, mutational status, AJCC staging, Ctn levels before surgery, CEA levels before surgery, Ctn levels after surgery and CEA levels after surgery were used as covariates. Correlation analyses were also performed for each pEV miRNA and the above-mentioned covariates for the validation cohort using GraphPad Prism version 8 (San Diego, California, USA). *p* values < 0.05 were considered statistically significant for all analyses.

## Results

### pEV miRNA and tissue expression profiles in discovery cohort

Plasma extracellular vesicles (pEVs) were isolated and characterized by evaluating the expression of EV markers. In detail, pEV expressed common EV markers (TSG101, CD63, CD81 and HSP70) in absence of the intracellular marker (Calnexin) (Supplementary Fig. 1). Circulating pEV miRNA profiles were obtained from MTC patients and control subjects (CTRL) along with miRNA profiles from paired MTC tumour samples.

**Fig. 2:**
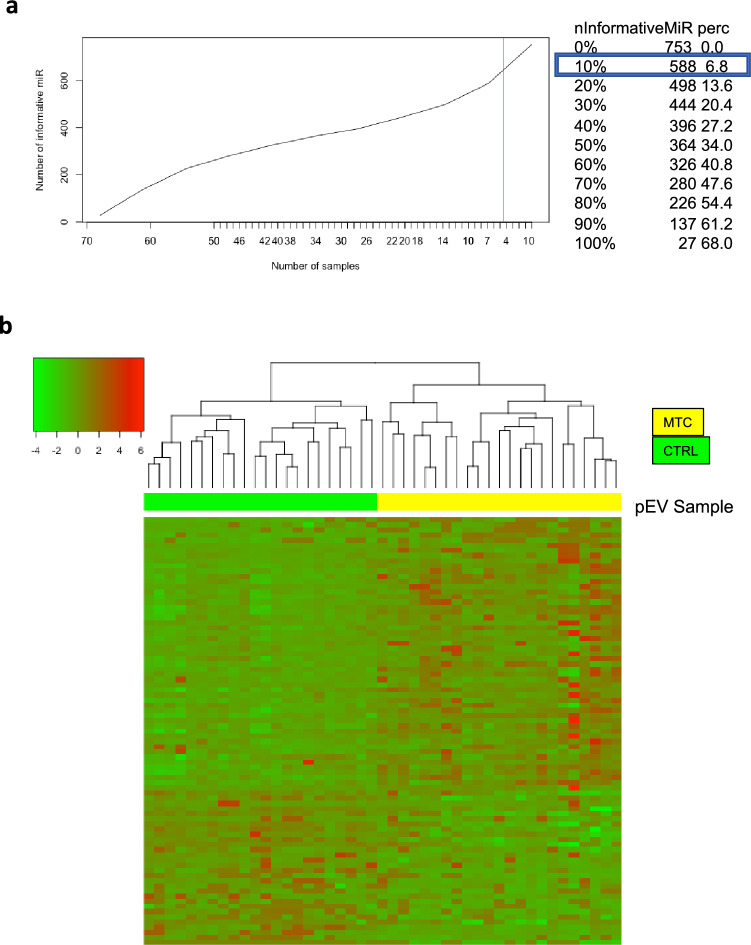
MiRNA profiles and differentially expressed circulating miRNAs in MTC. **a** Number of miRNAs detected in Medullary Thyroid Cancer (MTC) tumour and circulating samples. Number of pEV miRNAs detected in all samples with a Ct < 33 values. The red line indicates the chosen percentage of number of samples (10%) and the respective informative pEV miRNAs (*n* = 588). **b** Differentially expressed pEV miRNAs in MTC patients and control subjects (CTRL). Heatmap of 79 DE pEV miRNAs, of which 26 up-regulated and 53 down-regulated in MTC (yellow) compared to CTRL (green) subjects

Comparing the circulating miRNA profiles from MTC patients and CTRLs, 555 pEV miRNAs were detected in both groups. Moreover, 588 miRNAs were detected in at least 10% of MTC tumour and MTC paired circulating samples (Fig. 2A).

Differential expression analyses of circulating pEV miRNA profiles in MTC patients compared to CTRL resulted in 79 differentially expressed (DE) pEV miRNAs, of which 26 up-regulated and 53 down-regulated (Table 3).

**Table 3:** Differentially expressed pEV miRNAs in MTC and CTRL subjects

miRNA	Fold change (FC)	*p* value	Expression in MTC patients
hsa-miR-101-5p	− 4.46	2.94E-05	Up-regulated
hsa-miR-1183	− 3.12	4.82E-02	
hsa-miR-16-5p	− 1.34	9.24E-05	
hsa-miR-187-3p	− 3.90	5.04E-03	
hsa-miR-18b-5p	− 1.57	8.96E-05	
hsa-miR-198	− 1.69	3.87E-02	
hsa-miR-223-3p	− 1.18	1.09E-03	
hsa-miR-224-5p	− 1.51	2.84E-02	
hsa-miR-26b-5p	− 1.26	4.13E-06	
hsa-miR-301a-3p	− 2.50	1.21E-02	
hsa-miR-301b-3p	− 2.34	2.12E-02	
hsa-miR-324-3p	− 1.09	2.38E-02	
hsa-miR-370-3p	− 2.59	1.46E-02	
hsa-miR-373-3p	− 3.32	1.66E-03	
hsa-miR-412-3p	− 5.46	7.76E-04	
hsa-miR-450b-3p	− 1.25	3.77E-02	
hsa-miR-451a	− 2.17	4.07E-04	
hsa-miR-454-3p	− 2.47	4.10E-02	
hsa-miR-495-3p	− 1.28	2.92E-02	
hsa-miR-513a-5p	− 2.36	1.13E-02	
hsa-miR-548d-3p	− 2.42	4.10E-02	
hsa-miR-548j-5p	− 3.95	3.09E-02	
hsa-miR-574-3p	− 1.98	1.13E-04	
hsa-miR-593-3p	− 1.02	3.65E-03	
hsa-miR-654-3p	− 6.51	2.14E-04	
hsa-miR-770-5p	− 5.13	1.56E-04	
hsa-let-7d-3p	1.27	4.63E-08	Down-regulated
hsa-let-7f-1-3p	3.07	4.38E-03	
hsa-let-7i-3p	1.70	1.05E-05	
hsa-miR-100-5p	2.31	2.07E-06	
hsa-miR-10a-5p	1.08	1.66E-02	
hsa-miR-10b-5p	1.08	1.71E-04	
hsa-miR-1180-3p	1.15	6.58E-04	
hsa-miR-1208	3.60	1.93E-02	
hsa-miR-125b-5p	1.38	3.86E-07	
hsa-miR-130b-5p	1.66	1.46E-02	
hsa-miR-133a-3p	2.11	5.80E-04	
hsa-miR-183-5p	3.00	1.13E-02	
hsa-miR-188-5p	2.29	2.58E-06	
hsa-miR-193a-5p	2.32	7.41E-07	
hsa-miR-193b-3p	2.27	1.16E-03	
hsa-miR-200a-3p	1.46	4.26E-06	
hsa-miR-215-5p	2.39	8.07E-06	
hsa-miR-217	3.96	2.68E-02	
hsa-miR-222-3p	1.21	5.29E-05	
hsa-miR-24-3p	1.58	8.04E-06	
hsa-miR-296-5p	3.12	4.47E-06	
hsa-miR-29b-2-5p	1.72	3.96E-04	
hsa-miR-29c-5p	1.06	3.75E-05	
hsa-miR-30a-5p	4.77	1.42E-02	
hsa-miR-320a	1.07	2.84E-06	
hsa-miR-325	1.54	6.43E-05	
hsa-miR-331-5p	3.89	1.86E-05	
hsa-miR-339-3p	1.22	3.05E-06	
hsa-miR-340-5p	1.01	3.05E-04	
hsa-miR-345-5p	1.18	2.08E-03	
hsa-miR-362-3p	1.84	6.64E-03	
hsa-miR-362-5p	2.15	7.30E-07	
hsa-miR-378a-3p	1.06	1.30E-06	
hsa-miR-423-5p	1.05	1.91E-05	
hsa-miR-450b-5p	4.95	9.16E-04	
hsa-miR-499a-5p	2.16	1.24E-04	
hsa-miR-500a-3p	1.29	5.56E-06	
hsa-miR-500a-5p	1.58	9.53E-04	
hsa-miR-501-5p	1.69	5.28E-05	
hsa-miR-532-3p	1.43	1.03E-03	
hsa-miR-532-5p	1.94	1.29E-07	
hsa-miR-548e-3p	1.37	1.66E-03	
hsa-miR-548k	1.13	3.98E-02	
hsa-miR-570-3p	1.65	1.46E-02	
hsa-miR-576-5p	1.61	4.98E-06	
hsa-miR-624-5p	1.22	4.31E-04	
hsa-miR-627-5p	1.10	1.10E-02	
hsa-miR-660-5p	1.91	3.64E-07	
hsa-miR-769-5p	2.29	1.06E-05	
hsa-miR-885-5p	1.86	1.36E-03	
hsa-miR-941	1.45	1.66E-02	
hsa-miR-96-5p	1.38	1.63E-04	
hsa-miR-99b-5p	1.41	5.17E-07	

Hierarchical clustering of the DE pEV miRNAs clearly separated the subjects in two distinct clusters that segregate MTC and CTRL samples (Fig. 2B).

### Identification of pEV miRNA signature for MTC diagnosis

To identify MTC circulating biomarkers among DE pEV miRNAs, we combined two strategies, first by performing a least absolute shrinkage and selection operator (LASSO) logistic regression analysis using as input the 79 DE pEVs and then by focussing only on pEV miRNAs which resulted up-regulated in MTC patients and applying expression and statistical significance criteria. In detail, the criteria consisted of selecting pEV miRNAs with high expression in MTC patients (Ct < 24), miRNA detection in at least 70% of the samples in each subject group and exclusion of those miRNAs expressed at low levels (Ct > 27) in MTC tumour samples. These criteria were applied so that miRNAs up-regulated in pEV (Ct < 24) while at low levels in MTC tissues (Ct > 27) were not taken into consideration, since we deemed them less likely to be cancer cell-related and useful in forecast of their possible application to the clinical setting.

The LASSO logistic regression analysis allows to fit a generalised model using penalized maximum likelihood thus identifying a group of features that can characterise a condition.

**Fig. 3:**
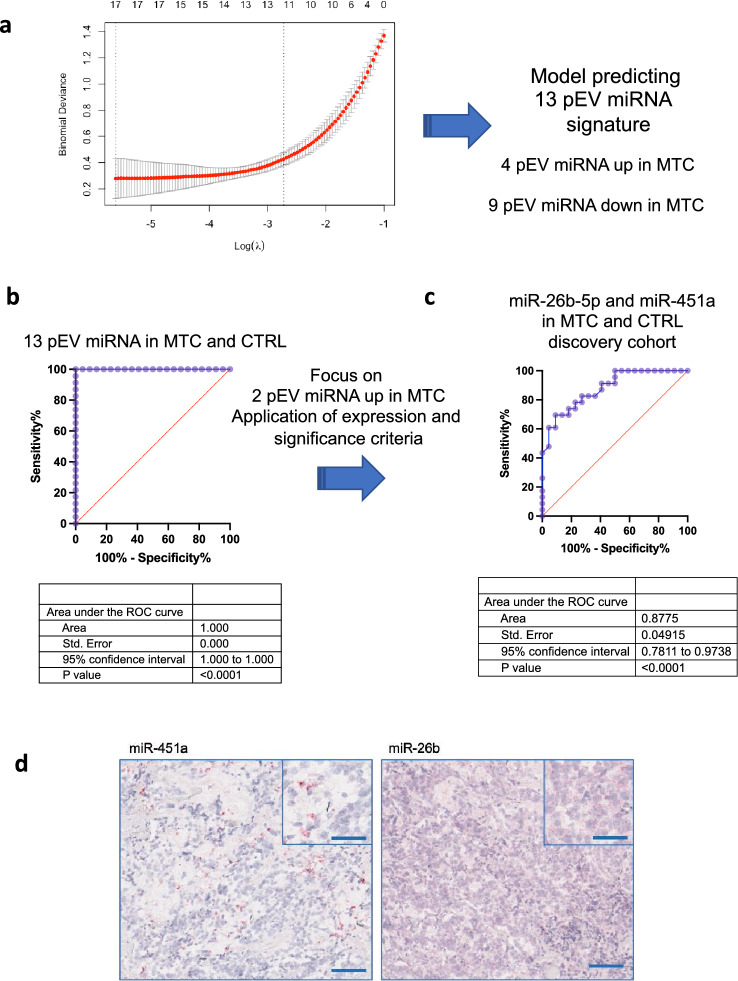
LASSO predictive model identifies a 13 pEV miRNA MTC diagnostic signature. **a** Parameter selection in LASSO regression and 13 pEV miRNA signature. **b** ROC curve of 13 pEV miRNAs (AUC = 1.00 *p* < 0.0001) and application of expression and significance criteria identifying pEV miR-26b-5p and miR-451a as putative biomarkers. **c** ROC curve of 2 pEV miRNAs, miR-26b-5p and miR-451a, (AUC = 0.8775, *p* < 0.0001). Blue line = sensitivity, red line = identity. **d** Representative image of in situ hybridization (RNAscope^®^) showing the expression of miR-451a and miR-26b in MTC tissue samples (dot-like pattern staining). Magnification 10X, scale bar 100 μm; inset at 20X, scale bar 50 μm

LASSO logistic regression analysis was performed using the 79 DE pEVs (Table 3) as input. The LASSO predictive model identified a putative 13 pEV miRNA diagnostic signature that included 4 up-regulated and 9 down-regulated pEV miRNAs in MTC (Fig. 3A). The high diagnostic performance of the 13 pEV miRNA signature is demonstrated in Fig. 3B.

Since a molecule that is expressed at higher levels in patients will be detected more easily and is more suitable as potential biomarker, we focussed on the 4 pEV up-regulated miRNAs in MTC patients. Among the 4 pEV up-regulated miRNAs, 2 pEV miRNAs satisfied the significance criteria described above (Supplementary Table 2 and Fig. 3B).

The combined strategies allowed us to identify miR-26b-5p and miR-451a as two putative diagnostic biomarkers.

Next, we evaluated their discriminatory ability and ROC analysis of miR-26b-5p and miR-451a, using the average expression of these two miRNAs, which resulted in an AUC equal to 0.87, illustrating the significant diagnostic performance of the two pEV miRNAs (Fig. 3C). Finally, in situ experiments were performed reporting miR-26b-5p and miR-451a expression in MTC tumour samples (Fig. 3D).

### miR-26b-5p and miR-451a expression levels before and after surgery

MTC patients from the discovery cohort underwent surgery and plasma samples from 10 patients were collected during follow-up. The patients were classified 3 months after surgery: 2 patients presented evidence of disease, while 8 were disease free. Evaluation of miR-26b-5p and miR-451a before and after surgery was performed in plasma samples from these 10 MTC patients.

**Fig. 4:**
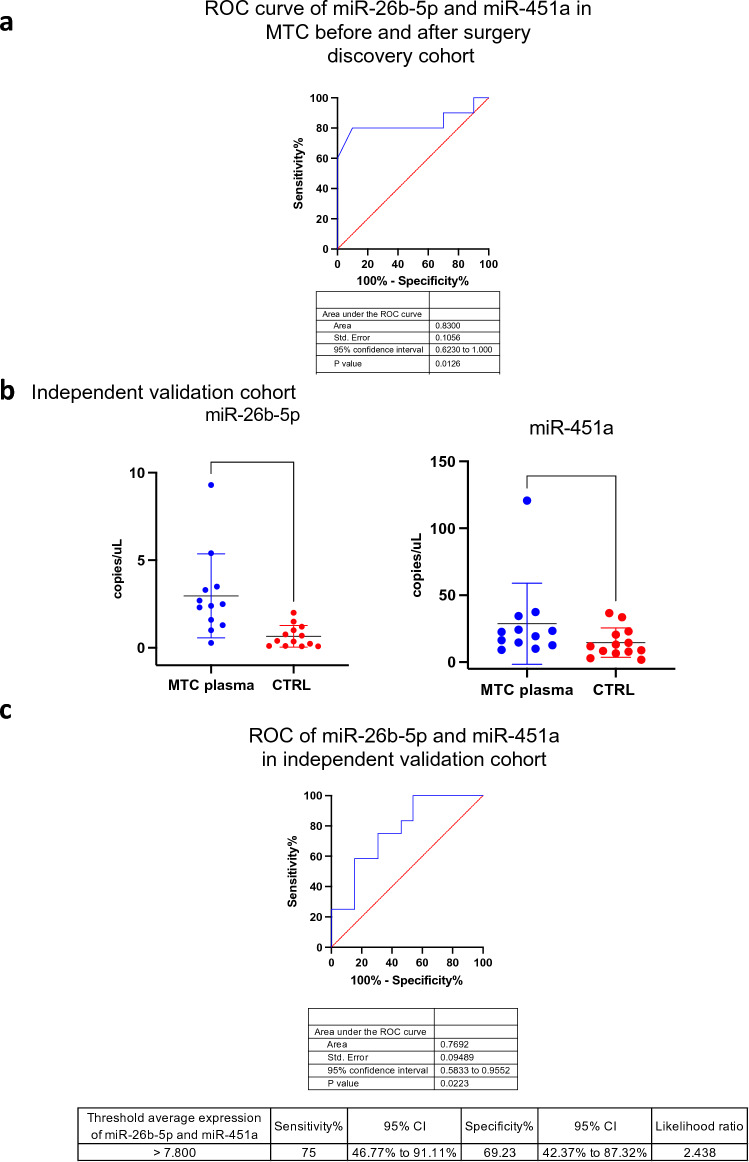
Validation of circulating miRNAs as diagnostic and monitoring biomarkers in MTC. **a** ROC curve of 2 pEV miRNAs in 10 discovery cohort MTC samples before and after surgery (AUC = 0.83 *p* = 0.0126). Blue line = sensitivity, red line = identity. **b** ddPCR copies/μL of miR-26b-5p and miR-451a pEV miRNAs in MTC patient (blue dots) and CTRL plasma samples (red dots). *p < 0.05, **p < 0.01, ***p < 0.001, ****p < 0.0001. **c** ROC curve of miR-26b-5p and miR-451a pEV miRNAs in an independent validation cohort of 12 MTC and 13 CTRL subjects (AUC = 0.769; *p* value = 0.0223). Blue line = sensitivity, red line = identity

With the aim to evaluate the pre and post-surgery significance of the two selected pEV miRNAs, we performed ROC curve analysis taking into account pEV miRNA levels before and after surgery, which resulted in an AUC equal to 0.83 (Fig. 4A). Correlation analyses of pEV miRNAs expression levels with Ctn levels after surgery and CEA levels after surgery did not report any significant results.

These results highlight the potential role of pEV miR-26b-5p and miR-451a as post-surgery biomarkers for MTC.

### Enrichment pathway analysis of miR-26b-5p and miR-451a

To gain insight into the putative role of miR-26b-5p and miR-451a in pathophysiological processes, an enrichment pathway analysis was performed.

Validated target genes of miR-26b-5p and miR-451a pEV miRNAs were queried and used as input for pathway enrichment analysis. The top twenty enriched pathways are reported in Supplementary Table 3, among which “Proteasome”, “Melanoma”, “Non-small cell lung cancer” and “Phosphatidylinositol signalling system” pathways that attracted our attention in the context of MTC. In detail, proteasome for the regulation of protein expression, melanoma and non-small cell lung cancer for their neuroendocrine nature and phosphatidylinositol signalling system since phosphatidylinositol 3-kinase/protein kinase B (PI3K/AKT) activation triggered by RET has been observed in MTC [28]. The enrichment pathway analysis indicates how the two pEV miRNAs could be associated with MTC disease status.

### Validation of miR-26b-5p and miR-451a as diagnostic biomarkers in an independent cohort

To validate the actual role of the two miRNAs of interest as diagnostic biomarkers, circulating pEV miR-26-5p and miR-451a expression were evaluated in an independent cohort of MTC patients and in control subjects (CTRL). DdPCR was performed to evaluate pEV miR-26-5p and miR-451a absolute expression levels. Statistically significant higher expression levels of both pEV miRNAs were observed in MTC patients compared to control subjects (Fig. 4B). ROC curve analysis with an AUC equal to 0.76 showed a good diagnostic performance (Fig. 4C).

Multivariate and correlation analyses of pEV miRNA levels with clinical data (sex, age, mutational status, AJCC staging, Ctn levels before surgery, CEA levels before surgery, Ctn levels after surgery and CEA levels after surgery) did not provide evidence of correlation with any of the above-mentioned clinical data of MTC patients.

In conclusion, these results validate, for the first time, circulating pEV miR-26b-5p and miR-451a as diagnostic and monitoring biomarkers in MTC.

## Discussion

MTC patients can have a good prognosis, provided that the disease is diagnosed and treated at an early stage without distant metastasis. MTC patients will benefit from biomarkers that will allow not only early diagnosis but an efficient monitoring during follow-up, permitting the timely application of appropriate personalized treatment plans. The presence of biomarkers that can be influenced by other parameters regardless of the disease status hampers patient management and can delay the application of the most appropriate treatment [29].

Here, we aimed at addressing this medical need by taking advantage of the non-invasive characteristics of liquid biopsy. Circulating pEV miRNAs play a fundamental role in cell–cell communication, while presenting the advantages of being associated to specific pathological conditions, easy to detect in blood samples and thus helpful in monitoring disease status in patients in follow-up studies.

We had the opportunity to evaluate miRNA from tumour tissue and liquid biopsy of MTC patients. Hierarchical clustering demonstrated how the differentially expressed pEV miRNAs characterise MTC patients. Potential diagnostic biomarkers were evaluated among the differentially and highly expressed pEV miRNAs in MTC patients, whose expression was also reported in the respective tumour samples. pEV miR-26b-5p and miR-451a were highly expressed in MTC patients and ROC curve analyses demonstrated their high diagnostic and post-surgery role (AUC equal to 0.87 and 0.83 respectively).

Enrichment pathway analysis highlighted how the target genes of these two pEV miRNAs are associated to different pathways of interest among which, pathways related to tumours such as melanoma and non-small cell lung cancer but also PI3K/AKT, whose activation plays an important role in MTC development and maintenance.

Since one of the aims of this study was the identification of diagnostic biomarkers for MTC, we tested the performance of pEV miR-26b-5p and miR-451a.

The diagnostic performance of pEV miR-26b-5p and miR-451a was validated in a second independent cohort of MTC patients and control subjects using ddPCR, a technology that allows absolute quantification. ROC analyses confirmed the ability of pEV miR-26b-5p and miR-451a to identify MTC disease status (AUC equal to 0.76).

Circulating miR-26b-5p and miR-451a expression levels have been investigated in different types of cancer. In detail, high expression of circulating miR-26b-5p was reported in esophageal squamous cell carcinoma and breast cancer [30, 31], while low circulating expression was described in lung adenocarcinoma [32]. High expression of circulating miR-451a was described in pancreatic cancer [33], while its low expression was reported in breast cancer and diffuse large B-cell lymphoma among others [34, 35].

Importantly, a literature review on circulating miR-26b-5p did not provide records with any studies investigating its expression in MTC or its role as a cancer biomarker. Circulating miR-451a high expression was reported in serum samples of 15 MTC patients compared to subjects with benign thyroid nodules and control subjects [21]. No investigation was conducted on its ability as a cancer biomarker since the authors focussed their attention on other serum miRNAs.

Of note, both miR-26b-5p and miR-451a have been reported to be commonly detected in serum and plasma samples and their expression levels to be affected by haemolysis [36–38]. Our choice to focus on pEV miRNAs was guided by the desire to further exclude any possible effect of haemolysis.

To our knowledge, this is the first study reporting circulating pEV miRNA expression patterns in MTC patients.

The collaboration of different centres allowed the collection and analysis of MTC samples, that due to the rarity of the tumour is understandably not easily achievable. The use of pEV miR-26b-5p and miR-451a as monitoring biomarkers was based on the classification of patients 3 months after surgery in patients with evidence of disease and disease-free patients. These results surely need further investigation and validation in a larger cohort and with specific focus on possible correlation and/or integration with clinical markers of persistent/recurrent disease from other research groups. Of note, the validation of the two pEV miRNAs in a second independent cohort through ddPCR, a technology currently in use in hospitals throughout the world, can offer two novel diagnostic biomarkers, miR-26b-5p and miR-451a, as a tool readily applied to the clinic. Altogether, the results of the study can provide clinicians a most needed tool for MTC diagnosis and patient management.

### Supplementary Information

Below is the link to the electronic supplementary material.

Supplementary file1 (PDF 209 kb) Characterization of pEVs by western blot analysis of common extracellular vesicle markers (HSP70, TSG101, CD63 and CD81) and cell organelle (Calnexin) in whole cell lysate (WCL) and EVs isolated from 6 MTC plasma and 6 CTRL samples. WCL was loaded as positive control for Calnexin

Supplementary file2 (PDF 210 kb)

Supplementary file3 (DOCX 16 kb)

Supplementary file4 (DOCX 17 kb)

Supplementary file5 (DOCX 16 kb)

## Data Availability

Source data are provided with this paper. All other data supporting the findings of the study are available from the corresponding author upon request.

## Abbreviations

MTC: Medullary thyroid carcinoma (MTC)
Ctn: Calcitonin
MEN: Multiple endocrine neoplasia
RET: Rearranged during transfection
FNA: Fine needle aspiration
CEA: Carcinoembryonic antigen
EVs: Extracellular vesicles
miRNAs: MicroRNAs
pEVs: Plasma extracellular vesicles
CTRL: Control samples
FF: Fresh-frozen
FFPE: Formalin-fixed paraffin-embedded
RT: Room temperature
Ct: Cycle threshold
DE: Differentially expressed
LASSO: Least absolute shrinkage and selection operator
ROC: Receiver operating characteristics
ddPCR: Droplet digital PCR
AUC: Area under the ROC curve
