# Correction: Circulating miR‑26b‑5p and miR‑451a as diagnostic biomarkers in medullary thyroid carcinoma patients

**DOI:** 10.1007/s40618-023-02172-7

**Published:** 2023-08-10

**Authors:** Z. M. Besharat, S. Trocchianesi, A. Verrienti, R. Ciampi, S. Cantara, C. Romei, C. Sabato, T. M. R. Noviello, A. Po, A. Citarella, F. P. Caruso, I. Panariello, F. Gianno, G. Carpino, E. Gaudio, M. Chiacchiarini, L. Masuelli, M. Sponziello, V. Pecce, T. Ramone, F. Maino, F. Dotta, M. Ceccarelli, L. Pezzullo, C. Durante, M. G. Castagna, R. Elisei, E. Ferretti

**Affiliations:** 1 Department of Experimental Medicine, Sapienza University of Rome, Viale Regina Elena 324, 00161 Rome, Italy; 2 Department of Molecular Medicine, Sapienza University of Rome, 00161 Rome, Italy; 3 Department of Translational and Precision Medicine, Sapienza University of Rome, 00161 Rome, Italy; 4 Endocrine Unit, Department of Clinical and Experimental Medicine, University of Pisa, 56126 Pisa, Italy; 5 Department of Medical, Surgical and Neurological Sciences, University of Siena, 53100 Siena, Italy; 6 Biogem Scarl, Istituto di Ricerche Genetiche “Gaetano Salvatore”, 83031 Ariano Irpino, Italy; 7 Department of Electrical Engineering and Information Technology, University of Naples Federico II, 80138 Naples, Italy; 8 Thyroid Surgical Unit, IRCCS Fondazione G. Pascale, 80131 Naples, Italy; 9 Department of Radiological, Oncological and Anatomo-Pathological Sciences, Sapienza University of Rome, 00161 Rome, Italy; 10 Department of Anatomical, Histological, Forensic Medicine and Orthopedics Sciences, Sapienza University of Rome, Rome, Italy; 11 Tuscany Centre for Precision Medicine (CReMeP), 53100 Siena, Italy

**Correction: Journal of Endocrinological Investigation** 10.1007/s40618-023-02115-2

The 8th affiliation has been published incorrectly in the original publication. The complete correct affiliation should read as follows.

Thyroid Surgical Unit, IRCCS Fondazione G. Pascale, 80131 Naples, Italy

The original article has been corrected.

