# Supplementary figures and images for: Circulating miR-26b-5p and miR-451a as diagnostic biomarkers in medullary thyroid carcinoma patients
Source: J Endocrinol Invest. 2023 Jun 7;46(12):2583–99. doi: 10.1007/s40618-023-02115-2 (PMC10632281; doi:10.1007/s40618-023-02115-2)

# Supplementary Figure 1

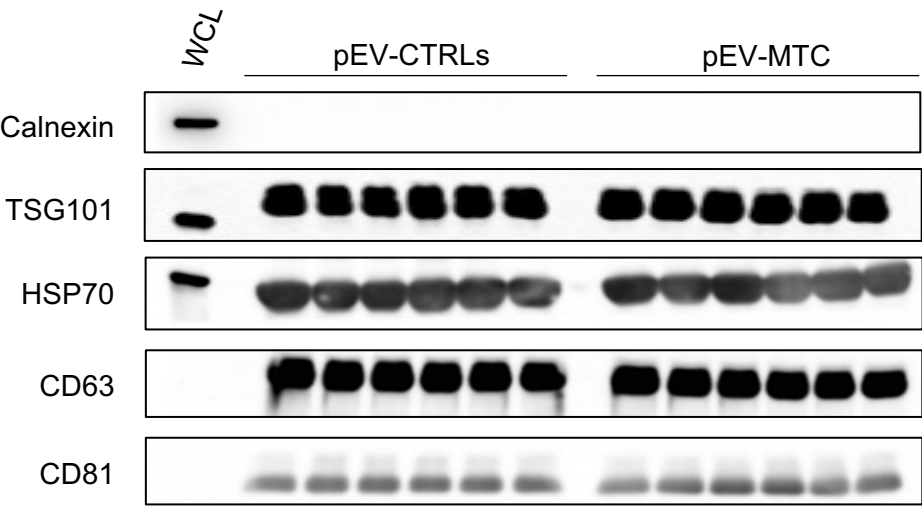

Supplement: Supplementary file 1 — Supplementary file1 (PDF 209 kb) Characterization of pEVs by western blot analysis of common extracellular vesicle markers (HSP70, TSG101, CD63 and CD81) and cell organelle (Calnexin) in whole cell lysate (WCL) and EVs isolated from 6 MTC plasma and 6 CTRL samples. WCL was loaded as positive control for Calnexin [file 40618_2023_2115_MOESM1_ESM.pdf]

Supplementary Figure 1 - full scan

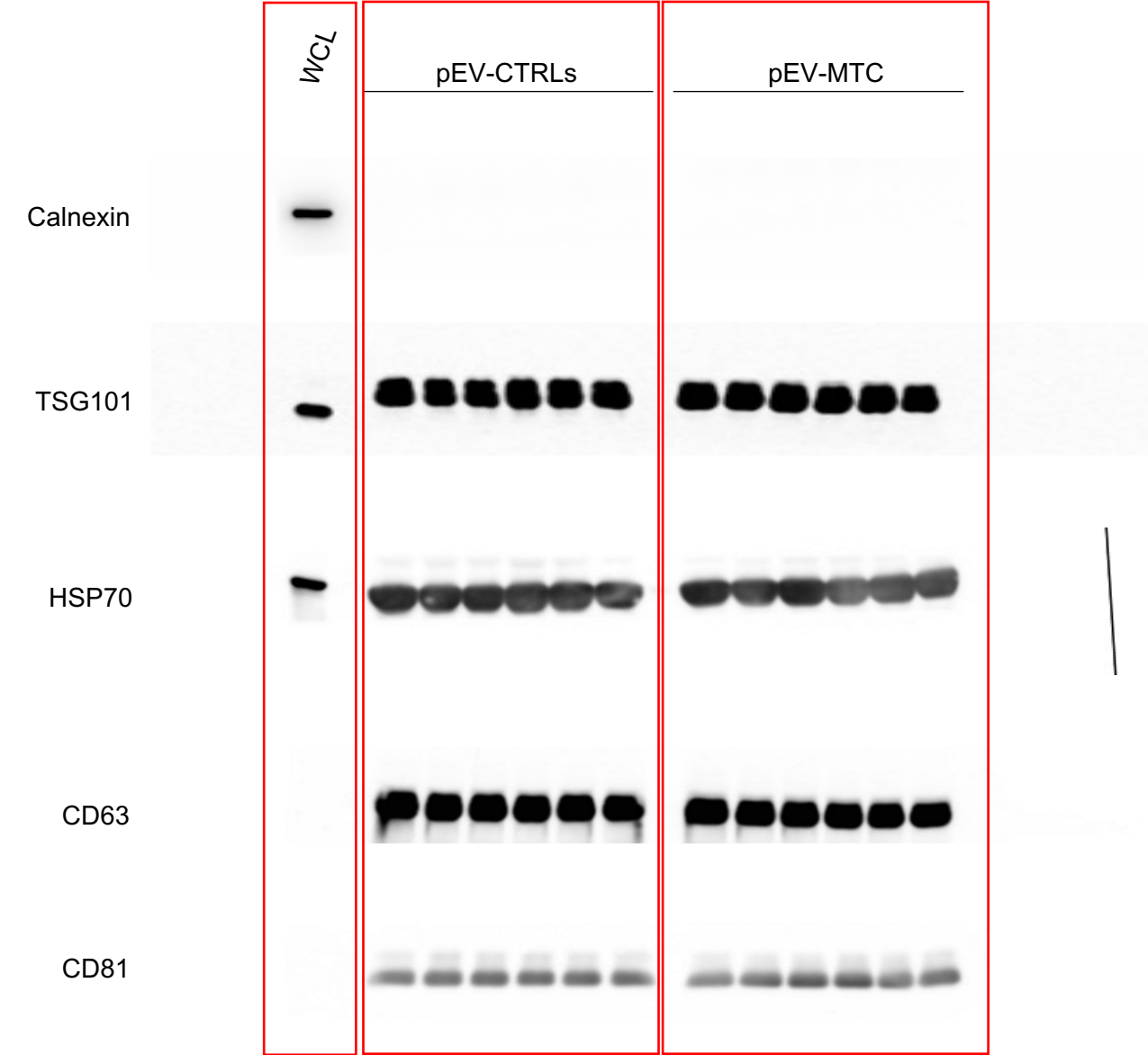

Supplement: Supplementary file 2 — Supplementary file2 (PDF 210 kb) [file 40618_2023_2115_MOESM2_ESM.pdf]
