# Supplementary material for: Circulating miR-26b-5p and miR-451a as diagnostic biomarkers in medullary thyroid carcinoma patients
Source: J Endocrinol Invest. 2023 Jun 7;46(12):2583–99. doi: 10.1007/s40618-023-02115-2 (PMC10632281; doi:10.1007/s40618-023-02115-2)
Supplement: Supplementary file 3 — Supplementary file3 (DOCX 16 kb) [file 40618_2023_2115_MOESM3_ESM.docx]

| **Supplementary Table 1. Characteristics of Control subjects (CTRL) of the discovery and validation cohorts.** | | | | | |
| --- | --- | --- | --- | --- | --- |
|  |  |  |  |  |  |
| **Control subjects (CTRL) characteristics of the discovery cohort.** | | | | | |
|  |  |  |  |  |  |
|  | CTRL subjects (n=22) | | |  |  |
|  | Sex | Male | n=10 |  |  |
|  |  | Female | n=12 |  |  |
|  | Age | Mean | 46.23 years |  |  |
|  |  | Median | 50.5 years |  |  |
|  |  | Range | 23-77 years |  |  |
|  |  |  |  |  |  |
|  |  |  |  |  |  |
| **Control subjects (CTRL) characteristics of the validation cohort.** | | | | | |
|  |  |  |  |  |  |
|  | CTRL subjects (n=13) | | |  |  |
|  | Sex | Male | n=7 |  |  |
|  |  | Female | n=6 |  |  |
|  | Age | Mean | 47.14 years |  |  |
|  |  | Median | 45 years |  |  |
|  |  | Range | 28-67 years |  |  |
