# Supplementary material for: Circulating miR-26b-5p and miR-451a as diagnostic biomarkers in medullary thyroid carcinoma patients
Source: J Endocrinol Invest. 2023 Jun 7;46(12):2583–99. doi: 10.1007/s40618-023-02115-2 (PMC10632281; doi:10.1007/s40618-023-02115-2)
Supplement: Supplementary file 4 — Supplementary file4 (DOCX 17 kb) [file 40618_2023_2115_MOESM4_ESM.docx]

| **Supplementary Table 2. 13 pEV miRNAs and their expression levels in MTC tumour samples.** | | |  |  |  |  |  |
| --- | --- | --- | --- | --- | --- | --- | --- |
|  |  |  |  |  |  |  |  |
| miRNA | Ct Mean values in MTC pEV | Ct Mean values in MTC tumor samples |  |  |  |  |  |
| hsa-let-7d-3p | 25.09 | 25.53 |  |  |  |  |  |
| hsa-miR-100-5p | 31.98 | 29.37 |  |  |  |  |  |
| hsa-miR-101-5p | 28.44 | 30.36 |  |  |  |  |  |
| hsa-miR-125b-5p | 28.04 | 19.65 |  |  |  |  |  |
| hsa-miR-24-3p | 27.42 | 25.47 |  |  |  |  |  |
| **hsa-miR-26b-5p** | **23.15** | **23.27** |  |  |  |  |  |
| hsa-miR-325 | 30.30 | 33.34 |  |  |  |  |  |
| hsa-miR-331-5p | 36.67 | 36.99 |  |  |  |  |  |
| hsa-miR-339-3p | 29.05 | 31.65 |  |  |  |  |  |
| **hsa-miR-451a** | **15.43** | **26.50** |  |  |  |  |  |
| hsa-miR-654-3p | 32.95 | 33.75 |  |  |  |  |  |
| hsa-miR-769-5p | 33.76 | 34.06 |  |  |  |  |  |
| hsa-miR-99b-5p | 29.94 | 25.14 |  |  |  |  |  |
|  |  |  |  |  |  |  |  |
| Ct: Cycle threshold |  |  |  |  |  |  |  |
| In **bold** the miRNAs selected for further investigation, red corresponds to up-regulated  and blue to down-regulated pEV miRNAs in MTC compared to CTRL. | | | | | | | |
