# Supplementary material for: Circulating miR-26b-5p and miR-451a as diagnostic biomarkers in medullary thyroid carcinoma patients
Source: J Endocrinol Invest. 2023 Jun 7;46(12):2583–99. doi: 10.1007/s40618-023-02115-2 (PMC10632281; doi:10.1007/s40618-023-02115-2)
Supplement: Supplementary file 5 — Supplementary file5 (DOCX 16 kb) [file 40618_2023_2115_MOESM5_ESM.docx]

| **Supplementary Table 3. Enrichment analysis of miR-26b-5p and miR-451a.** | | | |
| --- | --- | --- | --- |
| KEGG pathway | #genes | #miRNAs | P-value |
| Biosynthesis of unsaturated fatty acids | 6 | 1 | 1.90E-04 |
| Mucin type O-Glycan biosynthesis | 8 | 1 | 3.52E-03 |
| Glycosaminoglycan biosynthesis - chondroitin sulfate / dermatan sulfate | 9 | 1 | 3.64E-03 |
| Proteasome | 15 | 2 | 6.97E-03 |
| Fatty acid metabolism | 15 | 1 | 2.07E-02 |
| Non-small cell lung cancer | 16 | 2 | 2.85E-02 |
| Colorectal cancer | 18 | 2 | 3.60E-02 |
| Glioma | 19 | 2 | 3.60E-02 |
| Inositol phosphate metabolism | 21 | 2 | 3.60E-02 |
| Melanoma | 21 | 2 | 3.60E-02 |
| Prolactin signaling pathway | 22 | 2 | 4.16E-02 |
| Phosphatidylinositol signaling system | 25 | 2 | 4.65E-02 |
| Small cell lung cancer | 27 | 2 | 4.91E-02 |
| p53 signaling pathway | 27 | 1 | 9.35E-07 |
| Hippo signaling pathway | 31 | 2 | 2.67E-04 |
| Hepatitis B | 35 | 2 | 3.64E-03 |
| Huntington's disease | 39 | 2 | 2.07E-02 |
| Protein processing in endoplasmic reticulum | 40 | 1 | 2.83E-02 |
| Viral carcinogenesis | 49 | 2 | 3.24E-02 |
